# Albuminous and Non-Albuminous Sputa

**Published:** 1910-04-02

**Authors:** 


### ALBUMINOUS AND NON-ALBUMINOUS SPUTA.

It is not often that sputum is tested chemically, but there is evidence to show that, in addition to what can be learned from microscopical tests, some useful information is afforded by chemical examination, especially by determinations of whether the sputum contains albumen or not.

The investigation is not much more difficult than it is in the case of urine. If the sputum is abundant, especially in cases of bronchiectasis, it may be possible to obtain clear fluid from it directly. In other cases a small quantity of sputum may be put into a little normal saline solution and either shaken or well stirred after the addition of acetic acid to coagulate the mucin, the mixture being then filtered and the filtrate tested for albumen in the ordinary way.

One of the main points that have been ascertained is that tuberculous sputum almost always contains albumen, even when no tubercle bacilli can be found. It seems that the absence of any pronounced quantity of albumen from the sputum of a doubtful lung case is a decidedly favourable sign. The converse is not true, for phthisis is by no means the only lesion of the lung that renders the sputum albuminous.

The cases of bronchitis associated with heart disease fall into two groups in this respect—those with actual inflammation of the bronchial tubes and non-albuminous sputum, and those with no true bronchitis, but rather a passive exudate and albuminous sputum. It would be interesting to investigate this point further to see whether there is any difference in the prognosis in the two groups. When patients suffering from acute or chronic nephritis develop a cough which is regarded as bronchitic the sputum usually contains albumen, the reverse of what is the case in simple bronchitis. Of acute conditions, it may be noted that both lobar and broncho-pneumonia give rise to albuminous sputum.

